# A Study on Object Detection Performance of YOLOv4 for Autonomous Driving of Tram

**DOI:** 10.3390/s22229026

**Published:** 2022-11-21

**Authors:** Joo Woo, Ji-Hyeon Baek, So-Hyeon Jo, Sun Young Kim, Jae-Hoon Jeong

**Affiliations:** 1 School of Software Engineering, Kunsan National University, Gunsan-si 54150, Republic of Korea; 2 Department of Electrical and Computer Engineering, University of Sungkyunkwan, Seoul 16419, Republic of Korea; 3 School of Mechanical Engineering, Kunsan National University, Gunsan-si 54150, Republic of Korea

**Keywords:** objection detection, YOLOv4, tram, autonomous driving

## Abstract

Recently, autonomous driving technology has been in the spotlight. However, autonomous driving is still in its infancy in the railway industry. In the case of railways, there are fewer control elements than autonomous driving of cars due to the characteristics of running on railways, but there is a disadvantage in that evasive maneuvers cannot be made in the event of a dangerous situation. In addition, when braking, it cannot be decelerated quickly for the weight of the body and the safety of the passengers. In the case of a tram, one of the railway systems, research has already been conducted on how to generate a profile that plans braking and acceleration as a base technology for autonomous driving, and to find the location coordinates of surrounding objects through object recognition. In pilot research about the tram’s automated driving, YOLOv3 was used for object detection to find object coordinates. YOLOv3 is an artificial intelligence model that finds coordinates, sizes, and classes of objects in an image. YOLOv3 is the third upgrade of YOLO, which is one of the most famous object detection technologies based on CNN. YOLO’s object detection performance is characterized by ordinary accuracy and fast speed. For this paper, we conducted a study to find out whether the object detection performance required for autonomous trams can be sufficiently implemented with the already developed object detection model. For this experiment, we used the YOLOv4 which is the fourth upgrade of YOLO.

## 1. Introduction

Along with eco-friendly energy, autonomous driving is the most popular technology in the transportation industry. Various companies are developing autonomous driving technology, which in turn is in progress with the advancement of artificial intelligence. Autonomous driving is being studied not only in the transportation field, but also in various fields from urban design to social acceptance. Autonomous driving is classified into level 5, but so far, level 5 autonomous driving has not been implemented. Autonomous driving of automobiles has been actively developed, but autonomous trams have not been studied as much. Although trams run on public roads such as automobiles, they have some obvious differences, as they use tracks, sensors, and steel wheels. Since trams run on an installed track, there are few control elements, but there are clear disadvantages in that they have fewer options in an emergency. Therefore, problems may arise when autonomous driving technology used in automobiles is applied to trams [1,2,3,4].

In the reference [5], it was argued that autonomous driving of the railway system should increase cognitive distance and performance through the appropriate fusion of other sensors. As a result of applying and testing various sensor fusion methods to the railway system, it was explained that it is necessary to improve accuracy by using deep learning. Additionally, to find out the current status of research on autonomous trams, we investigated previous studies. As a result of this investigation, we confirmed that research on the method to create a profile for braking and acceleration and research on finding the location coordinates of nearby objects through object detection were completed. In this research, we checked that YOLOv3 was used for object detection to find object coordinates. YOLOv3 is an artificial intelligence model to position bounding boxes and classify objects in an image. YOLOv3 is the third upgrade of YOLO based on CNN, which is one of the most famous object detection technologies. YOLOv3′s object detection performance is characterized by ordinary accuracy and fast speed. Due to these characteristics, the YOLO application model is widely used with a camera in object detection technology and research [6]. AI models in data processing and analysis are keep upgraded. In this reference [7], the article proposes a multimodal deep learning (MDL) model that use the several feature maps to analyze several images for the same object. The model achieved higher accuracy by using CNN to the feature maps that obtained by multi-modality learning (MML) and cross-modality learning (CML).

In this paper, we conducted a study to find out whether the object detection performance required for autonomous trams can be sufficiently implemented with the currently developed object detection model. For this experiment, we used the YOLOv4 object detection model and used the dataset MS COCO and BDD 100K. The YOLOv4 model has improvements in accuracy and speed from YOLOv3. In the experiment, by using the BDD 100K dataset, it was checked whether problems were caused by physical differences even if learned through images taken from cars. In addition, it was possible to confirm which dataset was more suitable by comparing it with the object detection model using the MS COCO dataset [8,9].

The paper is organized as follows. In Section 2, the basic descriptions of datasets and YOLOv4 are explained. Section 3 describes the experimental and evaluation methods, the overall experimental process, and the analysis of experimental results. The conclusions are summarized in Section 4.

## 2. Materials and Methods

### 2.1. Dataset

**Figure 1:**
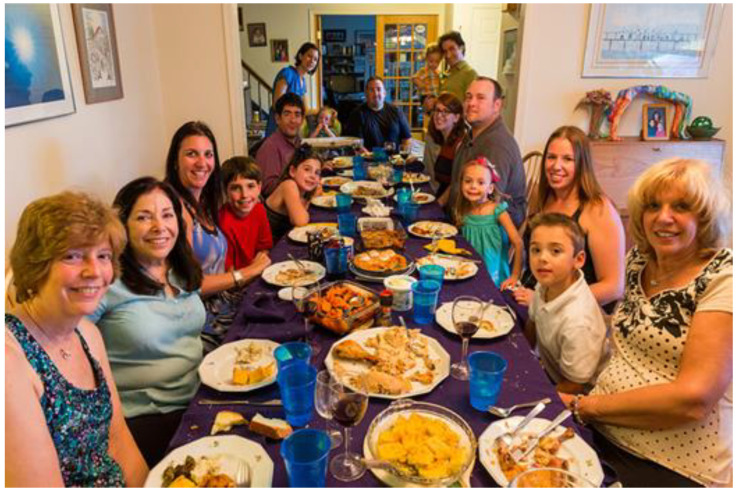
MS COCO dataset sample.

The MS COCO dataset is a representative dataset that tests object detection performance and is used in various competitions. This dataset has 80 classes and includes various images for each class. Unlike previously published image datasets (PASCAL VOC dataset, ImageNet dataset, etc.), the MS COCO dataset provides an image that includes multiple objects of various sizes. Conversely, when learning is performed using a dataset in which an object is mainly located in the center of an image, it is often difficult to recognize an object in a real environment. MS COCO, which learns from datasets with different sizes (especially if there are many small objects) and multiple objects, can perform object detection smoothly in real environments. Figure 1 is a sample of MS COCO datasets [10].

**Figure 2:**
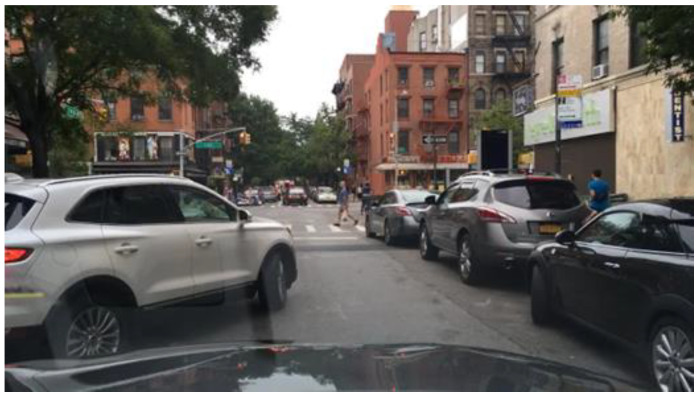
BDD 100K dataset sample.

The BDD 100K dataset is an image-based dataset created to recognize objects that can be variables for autonomous driving. This dataset mainly uses images captured by a camera installed in a vehicle and has 10 object classes that are mainly encountered while driving. Like the MS COCO dataset, the image of the BDD 100K dataset often has multiple objects of various sizes. Figure 2 is a sample of the BDD 100K dataset. The number of classes and data structure of the two datasets are summarized in Table 1 [11].

### 2.2. YOLOv4

YOLO is an object detection model first proposed by Joseph Redmon. He had developed YOLOv3. In this paper, we conducted an object detection experiment on a tram using YOLOv4, the fourth version of YOLO proposed by Alexey Bochkovskiy. YOLOv4 improves the speed and accuracy of YOLOv3. YOLOv3 can recognize objects at high speed, but the average precision is somewhat lower than other object detection models. YOLOv4 is the version that compensates for these shortcomings [8,9].

**Figure 3:**
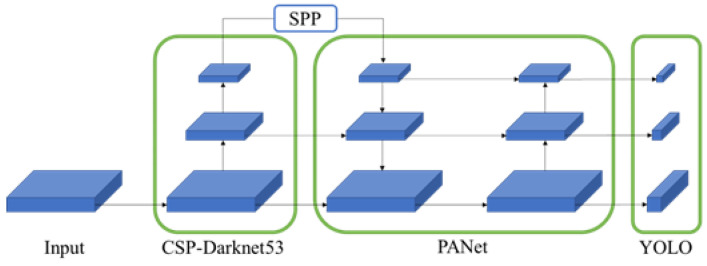
The architecture of YOLOv4.

The architecture of YOLOv4 consists of a backbone, detection neck, and detection head. The backbone is CSP-Darknet53. CSP-Darknet53 is a backbone model that improves the operation speed by applying CSP-DenseNet to Darknet53 used in YOLOv3. The detection neck uses SPP Block and PANet. SPP Block is used for the most downsized feature map of CSP-Darknet53. PANet performs upsizing and downsizing sequentially using the feature maps created through SPP Block and various levels of CSP-Darknet53. The detection head is the same as YOLOv3. Figure 3 shows the overall YOLOv4 architecture [9].

In YOLOv4, various techniques were used. The method can be divided into Bag of Freebies (BoF) and Bag of Specials (BoS). BoF refers to a learning method developed to improve performance without affecting inference time. BoS means a module or post-processing method that significantly improves accuracy even if the inference time slightly increases.

The BoF used in the backbone of YOLOv4 is CutMix, Mosaic, DropBlock, and Class Label Smoothing. CutMix and Mosaic are data augmentation techniques. CutMix uses two images, and Mosaic uses four images. CutMix is a learning method that cuts out some objects in the learning image and fills the space with objects in other images. Mosaic is a technique for learning by making four different images into one image. The image has a batch normalization effect. The Mosaic technique reduces a mini-batch size through batch normalization. DropBlock is a regularization technique that sets some areas of the feature map to 0 during training and excludes them from training. This works in principle similar to the learning method called Dropout, and it can prevent overfitting. Class Label Smoothing allows a label value that is usually 0 or 1 to have a value between 0 and 1. This is a technique designed to deal with possible incorrect labels. That is, this technique calibrates the model so as not to have overconfidence concerning the labels and normalizes the model [9,12,13,14].

The BoS used in the backbone of YOLOv4 uses the Mish activation function, Cross-Stage Partial connections (CSP), and Multi-input Weighted Residual Connections (MiWRC). The Mish activation function is very similar to the Swish activation function. As a feature, the gradient does not disappear because it has an unbounded function in the positive domain. It is advantageous for optimization because the overall output is smoothly connected. As the Mish activation function has a regularization effect because it works as a bounded function on negative outputs. CSP divides the characteristics map of the base layer and transmits only one side through the existing neural network. The other side is concatenated with the feature map output from the existing neural network. This can reduce the inference time as well as increase the accuracy. CSP also significantly reduces memory usage and bottleneck phenomenon [9,15,16].

The BoF used in YOLOv4′s Detector includes CIoU-loss, CmBN (Cross-Iteration mini–Batch Normalization), DropBlock, Mosaic, Self-Adversarial Training (SAT), Grid sensitivity elimination, using multiple anchors for single ground truth, a cosine annealing scheduler, hyperparameter optimization, and random training shapes. CIoU-loss is one of the loss calculation methods using IoU (Intersection over Union) as the loss. If only IoU is used as the loss, learning becomes impossible if the bounding boxes do not overlap. Therefore, CIoU-loss calculates the loss by including the distance and aspect ratio between the centers as well as the overlapping area. CmBN is a modified version of CBN (Cross-Iteration Batch Normalization) for YOLOv4. CBN is a technique for performing current batch normalization using statistics of batches used for past and present learning. This technique compensates for differences between past and present batches by applying statistics from past and present batches to the Taylor series. Unlike CBN, CmBN performs batch generalization using statistics of mini–Batches in a single batch. The DropBlock and Mosaic are the same as BoF used in the backbone. SAT is a new data augmentation technique proposed in YOLOv4 and proceeds in two steps. Step 1 modifies the image instead of training the artificial neural network through forward propagation. Step 2 is object detection learning that optimizes weights using the modified image. The grid sensitivity elimination is a proposed method to solve the problem of the equation for calculating the center position of a bounding box. This technique uses the sigmoid function to obtain the coordinates, and the absolute value of the input must be very large for the output of the sigmoid function to be 0 or 1. That is, there is a problem in that it becomes difficult to obtain accurate coordinates as the coordinates are closer to the boundary of the grid. It solves the problem by multiplying the sigmoid function output by a value greater than 1. In YOLOv4, if the IoU (ground truth, anchor) is higher than the IoU threshold, multiple anchors are used for the ground truth. This method is used to quickly obtain a bounding box close to the ground truth. The cosine annealing scheduler is one of the ways to plan how the learning rate will change. This prevents the learning from stagnation in the local minimum and helps the model to learn accurately at a high speed. The random training shapes mean training the object detection model with different image sizes. This generalizes object detector inputs and prevents overfitting [9,13,17,18,19].

The BoS used in the detector of YOLOv4 is the Mish activation function, SPP, SAM, PAN, and DIoU-NMS. The Mish activation function is the same as described in Backbone’s BoS. SPP is a method to make the output size the same regardless of the feature map with different sizes by performing pooling by multi-filter. In YOLOv4, SSP can use multiple sizes of the receptive field by using pooling with multiple sizes and can perform quick computation. This feature can distinguish important features of the context while maintaining the computation speed. SAM and PANet are slightly modified from the original versions. SAM outputs a feature map that is reduced in size to one feature map through a convolution on the feature map obtained through the max pooling and average pooling. This method has the advantage of efficiently transmitting important spatial-wise information with a small increase in inference cost. In YOLOv4, a convolution operation is performed instead of pooling in SAM. Therefore, the spatial-wise information transmitted by SAM becomes pointwise. PANet was proposed to fully utilize various levels of information. PANet transfers information from low-level to high-level without loss as much as possible, enabling more accurate localization. The modification of PANet in YOLOv4 is as follows: the change of shortcut connection from addition to concatenation. DIoU-NMS was used for post-processing in YOLOv4. DIoU-NMS is one of the methods of using IoU as the loss. DIoU is a method that calculates not only the degree of overlapping bounding boxes but also the distance between the bounding box of the real object and the center point of the predicted bounding box as the loss. NMS is a method of deleting the overlapping bounding boxes by judging that the same object is detected, leaving only the bounding boxes with high reliability [9,15,17,20,21,22].

## 3. Results

### 3.1. Methodology

**Figure 4:**
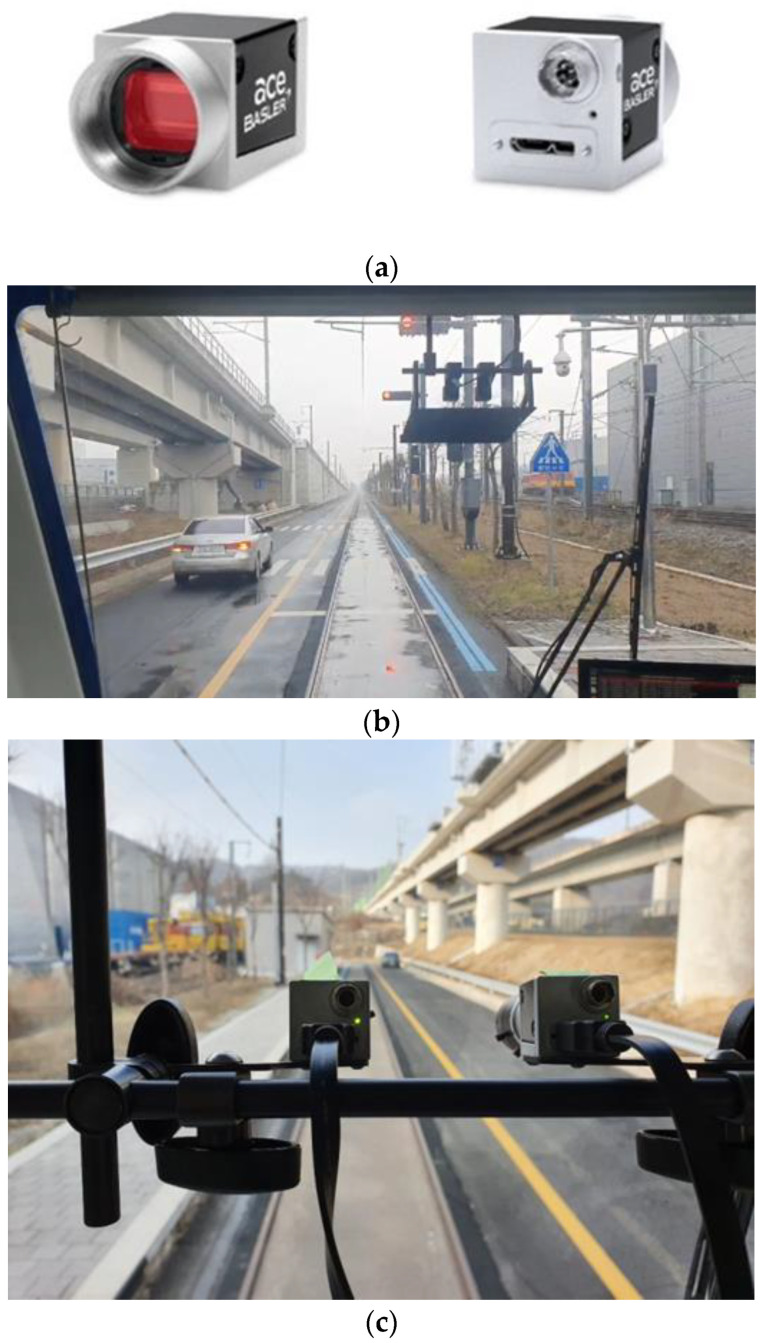
Camera installed inside tram: (**a**) camera (acA2000-165uc, Basler ace), (**b**) installed camera, and (**c**) installed camera (enlarged).

All object detection models presented in this paper were tested in the same environment. The specifications of the computer in which the experiment was conducted are RTX 2080 Ti (11 GB) GPU. The tram image was taken with a camera installed on the actual tram as shown in Figure 4. Both cameras are of the same model (acA2000-165uc, Basler ace). The recorded video is 2040 pixels wide and 1086 pixels high. The experiment was conducted at the Railway Research Institute (Osong, Korea).

In this paper, we compare the performance of the YOLOv4 model trained with MS COCO dataset and the YOLOv4 model trained with BDD 100K dataset. Since the two datasets have different numbers of classes, we edited the model structure for each dataset. After modifying the number of trainings to fit the datasets, we trained the models. However, we tested all other parts under the same conditions. We set the width and height to 416 × 416 for the input.

To compare these results, we identify the calculation results of mAP50, mAP75, FPS, the average IoU, Precision, Recall, and F1-Score in the two different object detection models trained with each dataset. Precision is an index that calculates precision by checking how many actual truths are included in the truth predicted by the model. The recall is the ratio of cases in which the model judges to be true among the actual truth. The F1-Score is an index to check whether the data of the model are unbalanced and are obtained using two indices, Precision and Recall. It is obtained by dividing the product of two indicators by the sum of the two indicators and multiplying by 2. IoU is an index indicating how much the bounding box predicted by the object detection model overlaps the bounding box that exists in the real object. This is a value obtained by dividing the overlapping portion of the two bounding boxes by the area occupied by the two bounding boxes on the image. Based on these indicators, we determine which dataset is more suitable for YOLOv4: the MS COCO dataset or the BDD 100K dataset. In mAP, AP stands for average precision. The area corresponding to the bottom of the Precision-Recall graph is defined as AP. The average value obtained by adding the AP values obtained for each class and dividing by the number of classes is called mAP. The value after mAP means the reliability threshold value of the bounding box for calculating mAP.

For qualitative evaluation rather than quantitative evaluation, we performed object detection in videos taken from real trams on both object detection models. For autonomous driving, we needed to measure the position of an object through the position of the bounding box when recognizing an object. Therefore, we checked which of the two models used in the experiment fits the location and size of the bounding box well with the real object. In addition, we analyzed the reliability of the bounding box and the ability to detect traffic-related objects through the videos obtained as the experimental results.

### 3.2. Methodology Experimental Results

**Figure 5:**
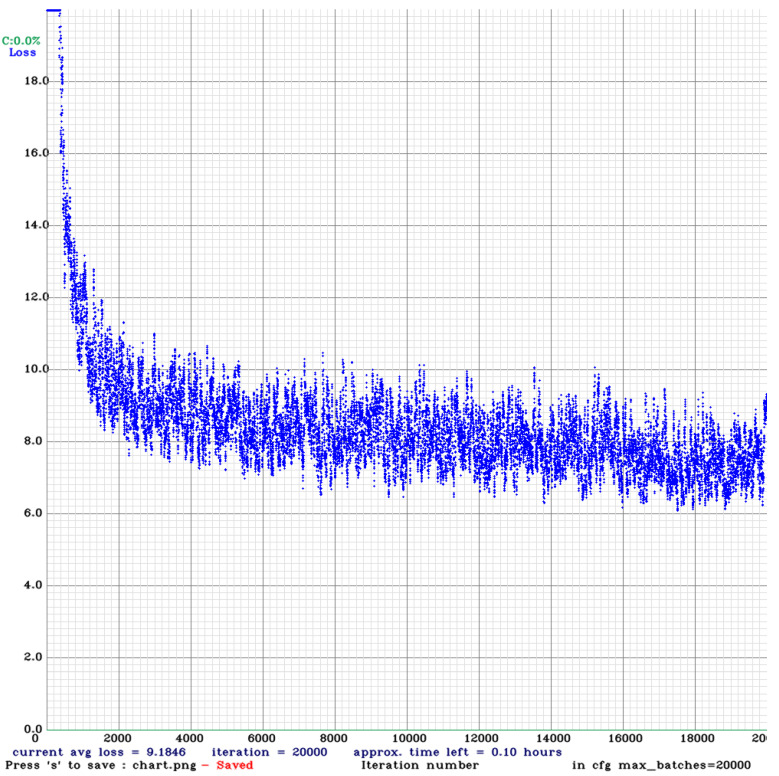
Loss graph while training YOLOv4 with BDD 100K dataset.

**Figure 6:**
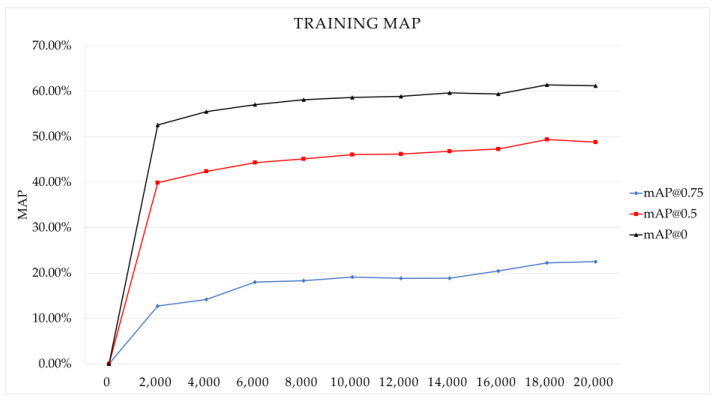
mAP graph while training YOLOv4 with BDD 100K dataset.

The BDD 100K dataset has 10 classes. As a result of training YOLOv4 with the structure and hyperparameters changed accordingly, we were able to obtain the weight file. Figure 5 shows the records of the change in loss during training. Figure 6 is the mAP recorded every 2000 iterations during training. As shown in Figure 5 and Figure 6, we have properly trained at a point where the loss is sufficiently reduced, the accuracy is no longer increasing, and it stagnates.

Table 2 shows the test results of the YOLOv4 model. The table includes mAP50, mAP75, and FPS. Table 3 indicates the detailed records of the test results according to the mAP of the BDD 100K dataset. As shown in Table 2, The YOLOv4 model trained on the BDD 100K dataset has mAP50 of 48.79% and mAP75 of 22.50%. The model trained with the MS COCO dataset has mAP50 of 62.80% and mAP75 of 44.30%. In comparison, the YOLOv4 model trained on the MS COCO dataset has better results. BDD 100K as shown in Table 3, as a result of training with the BDD 100K dataset, the average IoU is 47.08% at mAP50, and the average IoU is 32.40% at mAP50. At mAP50, Precision, Recall, and F1-Score are all equal to 0.61. At mAP75, Precision is 0.38, and Recall and F1-Score are 0.37.

The speed of YOLOv4 is compared by FPS (Frames per Second), which indicates the number of frames per second. Looking at Table 2, as a result of the experiment, the MS COCO model was 38.6 FPS and the BDD 100K model was 50.3 FPS. The 11.7 FPS difference is a fairly big one. Since the BDD 100K dataset has few classes, the number of output map channels in YOLOv4 is different. When trained with the MS COCO dataset, the final feature map of the YOLOv4 model has 255 channels. The 255 channels can be divided into 3 output information groups has 85 channels. In the group of 85 channels, two represent the coordinates of the object, two represent the sizes of the objects, one represents the confidence score, and the remaining 80 represent the classes of the objects. Therefore, in the case of the BDD 100K dataset, 45 channels are used, which is 210 fewer channels than the MS COCO dataset.

**Figure 7:**
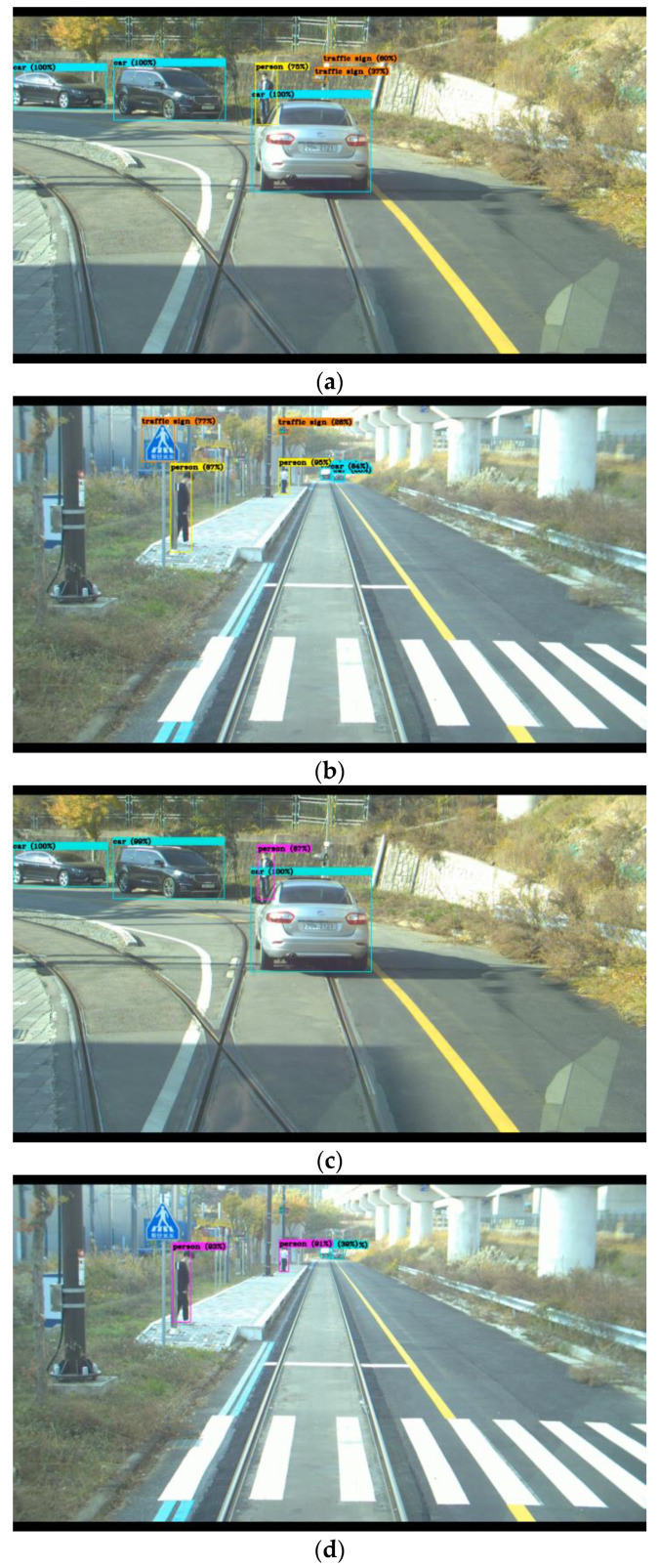
Experimental Video of Test Bed: (**a**) YOLOv4 Trained on BDD 100K Dataset (case1), (**b**) YOLOv4 Trained on BDD 100K Dataset (case2), (**c**) YOLOv4 Trained on MS COCO Dataset (case1), and (**d**) YOLOv4 Trained on MS COCO Dataset (case2).

Although mAP indicates the accuracy of the bounding box, it may be different in a tram environment. Therefore, we compared the two YOLOv4 models by using videos taken with the tram cameras. As a result, we confirmed that both object detection models recognize most of the learning objects. However, since we performed the supervised learning method, they were inevitably unable to recognize the unlearned objects. As shown in Figure 7, The model (Figure 7a,b) trained with the BDD 100K dataset recognizes traffic signals. However, the model (Figure 7c,d) trained with the MS COCO dataset does not recognize the signals at all because it does not include their classes.

## 4. Discussion

Experiments and results in the above chapter were obtained by referring to the description of GitHub uploaded by the author of YOLOv4 [9,23]. As shown in the mAP graph of Figure 6, we can see that it peaks at the 18,000 Iteration when the IoU Threshold is 0.5 and 0 and decreases at the 20,000 Iteration. However, in the graph where the IoU Threshold is 0.75, the mAP is increased at 20,000 Iterations. This means that learning is carried out properly before overfitting.

As shown in Table 2, we confirmed that the MS coco dataset has better mAP50 and mAP75 results than the BDD 100K dataset by analyzing the test results of each dataset. Based on the results, we analyzed that the MS COCO dataset is more suitable for the YOLOv4 model than the BDD 100K dataset. On the other hand, the model trained with the BDD 100K dataset is 11.7 FPS faster than the model trained with MS COCO. This difference occurs because the number of classes is small (BDD 100′s class: 10). In Table 3, when the IoU threshold of the YOLOv4 model trained on the BDD 100K dataset was changed from 0.5 to 0.75, the average IoU dropped by 14.68%P from 47.08% to 32.40%. The F1-Score was learned with a balanced score of 0.61 when the IoU threshold was 0.5 but was learned, relatively biased, as 0.37 when the IoU threshold was 0.75. This means that some classes are better learned, but others are not.

There was a difference between the two YOLOv4 models among the results of measuring objects in the video shot with the camera installed on the tram. Mainly, the BDD 100K dataset showed excellent performance in finding objects related to traffic. Comparing (a), (b), (c), and (d) in Figure 7, the model trained with BDD 100K detects the traffic sign, whereas the model trained with MS COCO, which does not detect its class, cannot detect it at all. MS COCO dataset can detect stop signs and traffic lights but can’t detect other traffic signs. On the other hand, the model trained with the MS COCO dataset had higher object detection confidence for humans. Looking at (a) and (c) of Figure 7, humans were detected equally, but 75% for BDD 100K and 87% for MS COCO. Looking at (b) and (d) of Figure 7, although humans are detected equally, BDD 100K has detection rates of 87% and 95%, and MS COCO has detection rates of 93% and 91%, exceeding 90%. It seems that this is the result of learning with various images about human objects.

## 5. Conclusions

Analyzing the results, we confirmed that the physical difference between the camera installed on the tram and the black box installed on the vehicle did not affect the object detection of the YOLOv4 model. YOLOv4 recognized the classes included in the dataset used for training well. Based on the results, we confirmed that the BDD 100K dataset has no advantage in autonomous driving over the MS COCO dataset except for the advantage of having a traffic object as a class. However, the BDD 100K dataset also includes a dataset on environmental changes according to weather and time. We believe that further experiments are needed to confirm these advantages. There is also a problem that vehicles, signs, and traffic lights are slightly different for each country. The reliability of the traffic sign located in front of the image in Figure 7b is 77%, which is lower than that of other objects in the images. In the future, when we create a dataset for autonomous driving for actual use, we plan to include more diverse classes and include datasets obtained from the traffic environment of each country for training. In addition, we are researching about activation function to make better object detection model. The function is targeting more improved activation function than Mish which used by YOLOv4. It is expected that an effective activation function can be created by strengthening the features mentioned in the paper introducing the Swish function.

## Figures and Tables

**Table 1:** Dataset information.

	Train	Validation	Test	Class
MS COCO	118,287	5000	40,670	80
BDD 100K	70,000	10,000	20,000	10

**Table 2:** mAP graph while training YOLOv4 with BDD 100K dataset.

	mAP@0.50	mAP@0.75	FPS
BDD 100K	48.79%	22.50%	50.3
MS COCO	62.80%	44.30%	38.6

**Table 3:** mAP graph while training YOLOv4 with BDD 100K dataset.

	Average IoU	Precision	Recall	F1-Score
mAP@0.50	47.08%	0.61	0.61	0.61
mAP@0.75	32.40%	0.38	0.37	0.37
